# Exome-Wide Association Study Reveals Several Susceptibility Genes and Pathways Associated With Acute Coronary Syndromes in Han Chinese

**DOI:** 10.3389/fgene.2020.00336

**Published:** 2020-04-09

**Authors:** Qiwen Zheng, Yan Zhang, Jie Jiang, Jia Jia, Fangfang Fan, Yanjun Gong, Zhi Wang, Qiuping Shi, Dafang Chen, Yong Huo

**Affiliations:** ^1^Department of Epidemiology and Biostatistics, School of Public Health, Peking University, Beijing, China; ^2^Department of Cardiology, Peking University First Hospital, Beijing, China

**Keywords:** exome-wide association study, acute coronary syndrome, control selection strategy, risk prediction tool, gene-based analysis, pathway-based analysis

## Abstract

Genome-wide association studies have identified more than 150 susceptibility loci for coronary artery disease (CAD); however, there is still a large proportion of missing heritability remaining to be investigated. This study sought to identify population-based genetic variation associated with acute coronary syndromes (ACS) in individuals of Chinese Han descent. We proposed a novel strategy integrating a well-developed risk prediction model into control selection in order to lower the potential misclassification bias and increase the statistical power. An exome-wide association analysis was performed for 1,669 ACS patients and 1,935 healthy controls. Promising variants were further replicated using the existing *in silico* dataset. Additionally, we performed gene- and pathway-based analyses to investigate the aggregate effect of multiple variants within the same genes or pathways. Although none of the association signals were consistent across studies after Bonferroni correction, one promising variant, rs10409124 at *STRN4*, showed potential impact on ACS in both European and East Asian populations. Gene-based analysis explored four genes (*ANXA7*, *ZNF655*, *ZNF347*, and *ZNF750*) that showed evidence for association with ACS after multiple test correction, and identification of *ZNF655* was successfully replicated by another dataset. Pathway-based analysis revealed that 32 potential pathways might be involved in the pathogenesis of ACS. Our study identified several candidate genes and pathways associated with ACS. Future studies are needed to further validate these findings and explore these genes and pathways as potential therapeutic targets in ACS.

## Introduction

Coronary artery disease (CAD) is the leading cause of death worldwide (Naghavi et al., 2017). In China, according to the National Health Committee’s epidemiological survey data of 2015, 136.1 per 100,000 deaths per year were estimated to be associated with CAD in urban areas and 144.8 per 100,000 deaths in rural areas. Hypertension, diabetes, dyslipidemia, obesity, and smoking are major risk factors for CAD (Khera and Kathiresan, 2017). Apart from these established environmental factors, genetic factors also play a pivotal role in determining an individual’s predisposition to CAD. Therefore, insights into the genetic basis of CAD might shed light on the identification of susceptible individuals, the exploration of disease pathogenesis, and the discovery of novel pharmaceutical targets for disease prevention and treatment.

During the past 10 years, several large-scale genome-wide association studies (GWAS) have successfully identified more than 150 loci with robust links to the risk of CAD (Erdmann et al., 2018; van der Harst and Verweij, 2018; Musunuru and Kathiresan, 2019). However, these variants only explained ∼20% of the observed disease variation, revealing the problem of “missing heritability”. Furthermore, most of these susceptibility variants are common variants with relatively small effects and are located within the intronic or intergenic regions. Their roles, therefore, remain difficult to interpret. Recent studies have drawn attention to the coding variants, which could directly provide biological and functional understanding of the etiologic mechanism. Using exome chip and whole-genome/exome sequencing techniques, several additional low-frequency coding variants associated with CAD have been detected, e.g., low density lipoprotein cholesterin-related genes (*PSCK9*, *LDLR*, and *NPC1L1*) and triglyceride-related genes (*APOA5*, *APCO3*, *LPL*, and *ANGPTL4*) (Khera and Kathiresan, 2017). These findings support the view that low frequency or rare variants in the coding regions may fill the missing heritability gap of CAD. Furthermore, owing to the differences in LD structure and MAF among different races and ethnicities, it is of great importance to conduct association analyses in non-European populations so as to detect novel loci associated with the risk of CAD.

Additionally, previous case-control GWAS usually selected a group of participants who were absent the disease of interest at enrollment as their control samples. However, one potential confounding issue was that these controls might not be truly “disease-free.” They might develop the disease of interest in the near future. As a result, the control selection strategy may lead to a misclassification bias and a subsequent loss in power. Such bias would be expected to be more pronounced in common diseases such as CAD (Mitchell et al., 2014). In order to address this issue, we proposed a novel strategy integrating a well-developed risk prediction model into control selection to lower the potential misclassification bias and increase the statistical power.

Consequently, using an improved control selection procedure, we conducted an EWAS in individuals of Chinese Han descent and replicated the promising variants, genes, and pathways in an existing *in silico* dataset. The objectives of the current study were: (i) to examine whether the known variants identified in Europeans are associated with CAD in the Chinese population; (ii) to explore novel genetic loci predisposing to CAD in Chinese subjects; and (iii) to investigate the aggregate effect of multiple variants within same genes or related pathways.

## Materials and Methods

### Study Population

Our study population comprised two cohorts taken from the ACS genetic study (Acute Coronary Syndrome Genetic Study) and PUUMA (Peking University-University of Michigan Study of Atherosclerosis) study. This study was approved by the Medical Ethics Committee of Peking University First Hospital and conducted in accordance with the Declaration of Helsinki. All participants were self-reported Han Chinese and provided written informed consent before taking part in this research.

The ACS genetic study is a prospective, observational, real-world practice cohort study comprising consecutive patients admitted to hospital for ACS within 48 h of symptom onset in China. Details of this study (NCT01964313) have been described elsewhere. In brief, a total of 1,803 patients were enrolled in the study at discharge. Baseline information, including demographics, medical history, disease characteristics, and treatment procedures was collected by the investigators. Information regarding occurrence of events, prescription status, other healthcare resource utilization, and self-reported quality of life was collected via telephonic interviews every 3 months during follow-up until 5 years after the ACS index event. ACS cases were defined as meeting at least one of the following diagnosis: STEMI, NSTEMI, and UA.

Non-CAD controls were selected from the PUUMA study. PUUMA is a large-scale project designed to study CVD and related traits in China (Ganesh et al., 2014; Tang et al., 2015). A total of 5,181 unrelated individuals were enrolled from a community-based cohort located in Beijing’s Shijingshan district. Details of the study have been reported previously (Cheng et al., 2016; Fan et al., 2016). Briefly, residents aged 40-years and above were invited to participate in this cohort by recruitment posters and telephone calls. All enrolled participants received a comprehensive baseline assessment of cardiovascular risk via face-to-face interviews, physical examinations, and laboratory tests. The structured questionnaire collected information regarding the sociodemographic status, diet, lifestyle, health behavior, and medical history. Interview questionnaire interview and anthropometric measurements were taken according to a standard operating procedure by the trained research staff. The participants’ medical, treatment, and family history of major cardiovascular risk factors were further checked against their medical records in community health centers. Individuals with unknown CVD status or previously diagnosed CVD were excluded from further involvement in the study.

### Genotype and Quality Control

All subjects in the ACS genetic study were genotyped using the Infinium HumanExome BeadChip V1.2 (Illumina, San Diego, CA, United States). The GenTrain version 2.0 in GenomeStudio V2011.1 (Illumina) was used to perform genotype calling. Quality control of the genotype calls in GenomeStudio was conducted under the best practice guidelines (Guo et al., 2014). Further quality control of the raw genotyping data was performed to filter unqualified genetic variants and samples (Supplementary Figure S1). A total of 134 ACS case samples were removed because they (i) had overall genotyping call rate < 95%; (ii) were biological relatedness, duplication, gender mismatch, or possible sample contamination; (iii) had an extreme heterozygosity rate more than 6 standard deviations from the mean. A total of 179,169 variants were excluded from subsequent analysis because they (i) had duplicate variants on the chip; (ii) were mitochondrial variants or were located on the X or Y chromosome; (iii) had a call rate < 95% or were monomorphic variants. For detection of ancestry and population stratification, a method based on principal-component analysis (PCA) was conducted using a panel of >20,000 independent common SNPs (MAF > 0.05). Seven outliers were removed from the analysis (Supplementary Figure S2A). Finally, 65,101 variants in 1,669 ACS cases were retained for further association analysis.

Genotyping and data quality control procedures of PUUMA study have been published previously (Tang et al., 2015). In short, all individuals were genotyped using the Asian Exomechip Infinium HumanExome BeadChip. Samples with low call rates (<99%), gender mismatch, biological relatedness, duplication, or possible sample contamination were removed. Marker-level quality control was performed to exclude variants with low cluster score, low call rate (<99.9%), monomorphic variants, and those that deviated from the Hardy–Weinberg equilibrium. After the previous quality control procedure, 4,458 non-CAD individuals with 129,306 variants were retained for further analysis.

In order to further verify the quality of samples, PCA was conducted using the independent common SNPs that survived the quality control procedures of both studies. There were no outliers examined from the study (Supplementary Figure S2B). Two biologically related samples were detected in the combined dataset, and we excluded one of the related samples in the control group. Consequently, 1,669 ACS cases and 4,457 controls with 50,023 overlapping qualified autosomal variants were retained for further analysis.

### Selection of Control

We integrated the risk prediction model into the control selection procedure in order to reduce the potential misclassification bias with the following steps. First, each healthy control sample received an assessment of future CVD risk according to the risk prediction model. Then, individuals in the high-risk group were excluded from subsequent association analysis. By stratifying individuals’ disease risk, we achieved a higher probability of selecting the truly “disease-free” controls, thereby lowering the potential misclassification bias and increasing the statistical power of our tests.

**FIGURE 1:**
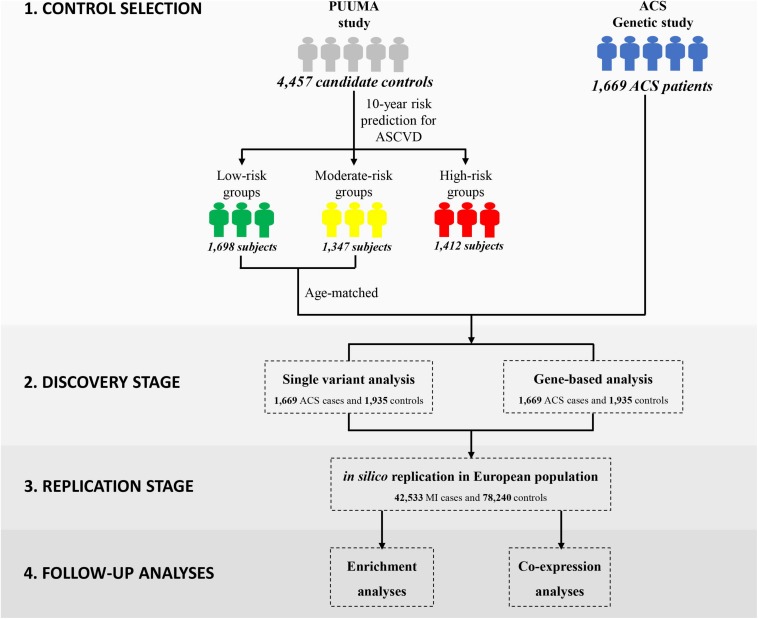
Overview of the study design and statistical analysis pipeline.

In this study, we employed the China-PAR equation (Prediction for ASCVD Risk in China) to evaluate each individual’s 10-year Atherosclerotic Cardiovascular Disease (ASCVD) risk (Figure 1) (Yang X. et al., 2016). In comparison to other well-known CVD risk evaluation tools derived from western samples (D’Agostino et al., 2008; Goff et al., 2014), the sex-specific China-PAR equation was an effective tool with good performance for the 10-year ASCVD risk prediction among Chinese populations. The China-PAR equation included previously identified major risk factors including age, treated or untreated systolic blood pressure, total cholesterol, high-density lipoprotein-cholesterol, current smoking (yes/no), and diabetes mellitus (yes/no) status. Furthermore, the model was improved by the inclusion of additional variables, including body mass index (BMI), waist circumference, geographic region (northern/southern China), urbanization (urban/rural), family history of ASCVD (yes/no), and interactions with age. The baseline characteristics of 4,457 candidate controls are presented in Supplementary Table S1. Applying the China-PAR equations resulted in groups of 1698, 1347, and 1412 subjects, stratified according to low (<5%), moderate (5–10%), and high (≥10%) 10-year ASCVD risk, respectively (Supplementary Figure S3) (Yang X. L. et al., 2016). Individuals in the high-risk group or subjects with unmatched age were excluded. Ultimately, 1,935 subjects were considered as truly “disease-free” controls and were included into subsequent exome-wide association analysis.

### Statistical Analysis

#### Single Variant Analysis

Assuming an additive genetic model, we performed single-variant tests by using logistic regression as implemented in PLINK 1.9 (Purcell et al., 2007). At the discovery stage, we carried out principal component analysis using samples that passed the quality control and selection procedure (Supplementary Figure S2C). The top two principal components were significantly (*P* < 0.05) associated with the outcome, and we therefore included them (together with age and gender) into the logistic regression model as covariates. Additionally, the Firth bias-corrected logistic likelihood-ratio test was also employed to assess the association results for rare or low-frequency variants. The attraction of this method is that it provides bias-reduction for small sample size as well as yields finite and consistent estimates even in case of separation (Wang, 2014). Based on the following criteria, we then selected 19 promising variants for further genotyping in the replication stage: (1) the single-variant association *P* < 0.0001; (2) variants were annotated as non-synonymous or splice sites. We defined statistical significance using the Bonferroni correction and set the exome-wide association significance threshold at 1 × 10^–6^ for single-variant analysis (0.05/50,023 variants tested). Quantile–quantile and Manhattan plots were generated by using R (V3.5.0, R Development Core Team). Regional plots were generated by using Locuszoom (Pruim et al., 2010).

To further assess the coding variants identified in the discovery stage, replication was carried out in an *in silico* meta-analysis of exome-chip studies of European descent involving 42,335 MI patients and 78,240 controls (Webb et al., 2017). Bonferroni correction was used to adjust for multiple comparisons (2.6 × 10^–3^, 0.05/19 variants tested). Variants were considered as successful replication based on the following criteria: (1) *P* < 2.6 × 10^–3^ in the replication cohort; (2) concordant direction of association in both discovery and replication populations.

#### Gene-Based Analysis

Given the different LD pattern between East Asians and Europeans, single markers may fail to be successfully replicated in our study. Gene- and pathway-based analysis, which synthesize information from multiple variants located in the same biological unit, might overcome the problem of genetic heterogeneity among different populations. Therefore, we performed gene-based analysis using the ‘SKAT’ R package: an unweighted combined multivariate collapsing burden test (CMC) (Li and Leal, 2008), and a sequence kernel association test (SKAT) (Wu et al., 2011). Variants were assigned to genes and functionally annotated using ANNOVAR (Wang et al., 2010). The gene-based analysis focused on missense or splicing variants with MAF less than 0.05 and predicted to be damaging (*n* = 22,729). Deleterious markers were defined as variants which were predicted to be damaging by CADD score (Kircher et al., 2014) or by at least two out of five functional prediction algorithms [SIFT (Kumar et al., 2009), Polyphen2 (Adzhubei et al., 2010), Mutation Taster (Schwarz et al., 2010), LRT (Chun and Fay, 2009), Mutation Assessor (Reva et al., 2011)]. We included only those genes for which two or more variants were present. Bonferroni correction was employed to define the significance threshold for gene-based analysis [*P* = 5.5 × 10^–6^, 0.05/(4,528 genes × 2 tests)].

The replication of gene-based analysis was performed in the meta-analysis dataset using MAGMA (de Leeuw et al., 2015). The 1000 Genomes Project Phase1 European reference population was used to estimate the LD between variants. Gene boundaries were defined as −35 kb upstream and +10 kb downstream, since transcriptional regulatory elements are likely to be contained within these intervals and thus, there is merit in capturing the signal of nearby SNPs that fall in the regulatory regions.

#### Pathway-Based Analysis

Pathway analysis was conducted using MAGMA (de Leeuw et al., 2015) to assess the enrichment of sets of functionally related genes (de Leeuw et al., 2015). Using results derived from the gene-based analysis, we calculated competitive gene set *P*-values based on the gene-wide *P*-values after accounting for gene size, gene density and minor allele count. Predefined gene sets were downloaded from the Molecular Signatures Database (Liberzon et al., 2011) (MSigDB version 6.2^1^), including KEGG, BioCarta, Reactome, and GO. We selected gene sets containing 11–200 genes, resulting in a total of 6,612 pathways. Bonferroni correction was applied for multiple testing correction in each gene set (*P* = 7.6 × 10^–6^).

#### Co-expression Analysis

As genes can influence each other through enhancement or hindrance, we may fail to reveal the true contribution of the detected signal if other functional-related genes are not taken into account. Those genes that co-express together tend to have similar biological functions. Identification of the biological functions behind the co-expression network will substantially increase our understanding of the biological mechanisms involved in disease pathogenesis. Genome-wide expression correlation analysis was performed to identify co-expressed disease-related genes in 49 MI patients from the GSE66360 gene expression microarray dataset (Muse et al., 2017). After Bonferroni correction, KEGG enrichment analysis of significantly co-expressed genes was conducted using the ‘clusterProfile’ R package (Yu et al., 2012).

## Results

### Single-Variant Association Analysis

Our general approach and analytical pipeline is outlined in Figure 1. Baseline characteristics of our study participants are shown in Table 1. After quality control and selection of control subjects, 3,604 Chinese Han subjects (1,669 cases and 1,935 age-matched controls with low or moderate risk for 10-year ASCVD) were available for the discovery-stage analysis. In the single-variant association analysis, the quantile–quantile plot revealed a good match between the distributions of the observed and expected *P*-values (Supplementary Figure S4). We did not observe evidence for inflation of test statistics for the association analysis, indicating a low possibility of false-positive associations resulting from population stratification. We examined the evidence for the previously reported GWAS loci (Supplementary Table S2). Of the 66 loci previously reported to be associated with CAD, 55 variants were tested in our study samples (48 directly genotyped and 7 with high LD proxies). Forty-two markers showed effects in the same direction as the previously reported studies. Amongst these, 10 SNPs (rs17465637, rs2023938, rs4977574, rs12413409, rs11042937, rs964184, rs17514846, rs46522, rs663129, and rs445925) also showed nominal significant association in our data. However, the effect estimates of rs17465637 at *MIA3*, rs2023938 at *HDAC9*, and rs11042937 at *MRVI1* demonstrated heterogeneity between our data and previous GWAS, which might partially arise from phenotypic differences and ethnic variations.

**TABLE 1:** Summary of study subject characteristics.

Characteristics		ACS patients (*N* = 1,669)	Controls (*N* = 1,935)
Age		61 (53–68)	55 (53–58)
Gender	Male	1286 (77.1%)	534 (27.6%)
	Female	383 (22.9%)	1401 (72.4%)
Smoking	Current-smoker	669 (40.1%)	290 (15%)
	Ex-smoker	272 (16.3%)	77 (4%)
	Never-smoker	728 (43.6%)	1568 (81%)
BMI		24.6 (22.8–26.8)	25.4 (23.3–27.6)
Hypertension		869 (52.1%)	637 (32.9%)
Type 2 diabetes		325 (19.5%)	179 (9.3%)
Type of ACS	UA	897 (53.7%)	
	STEMI	391 (23.4%)	
	NSTEMI	363 (21.7%)	

Of the 50,023 polymorphic SNPs examined, 19 non-synonymous variants were identified with *P* < 1.0 × 10^–4^ by single-variant association analysis (Supplementary Figure S5 and Supplementary Table S3). Of these, five variants passed the exome-wide significance threshold (rs117506953, *P* = 6.0 × 10^–26^; rs10409124, *P* = 6.6 × 10^–24^; rs73929373, *P* = 5.4 × 10^–10^; rs4127353, *P* = 3.1 × 10^–7^; rs149822831, *P* = 7.3 × 10^–7^). For an exome-wide study, independent validation is an effective approach to reduce false-positive associations. However, it is difficult to collect relatively large samples with both detailed clinical information and blood samples. Existing GWAS summary statistics can provide a highly cost-effective way for external validation, although potential association might be lost because of heterogeneity. Therefore, we further assessed the 19 promising variants in the meta-GWAS dataset and identified nine SNPs from the *in silico* exome-chip genotype data (Supplementary Table S4). None of the SNPs could be replicated after the Bonferroni correction, even if rs10409124 (Striatin 4, p.V568I) showed consistent association with the risk of ACS in both our study population and the meta-GWAS dataset (Table 2). Then, we conducted a stratification analysis for rs10409124 by age, gender, BMI, smoking, hypertension, and diabetes and did not find significant heterogeneity between different subgroups (Supplementary Table S5). Although this missense variant did not demonstrate being damaged according to SIFT or Polyphen2, it was close to the suggested deleterious threshold based on CADD algorithms (CADD score ≥ 12.37), which integrated more than 60 diverse annotations (Kumar et al., 2009; Adzhubei et al., 2010; Kircher et al., 2014).

**TABLE 2:** Markers associated with ACS identified by single-variant analysis.

Variant ID	Chr.	Major/Minor allele	Gene	Variant	Stage	OR (95%CI)	*P*-value
rs10409124	19q13.32	C/T	*STRN4*	c.1702G→A NM_013403.2	Discovery	3.87 (2.97–5.03)	6.6 × 10^–24^
					Replication	1.34 (1.03–15.8)	2.5 × 10^–2^

### Gene-Based Analysis

**FIGURE 2:**
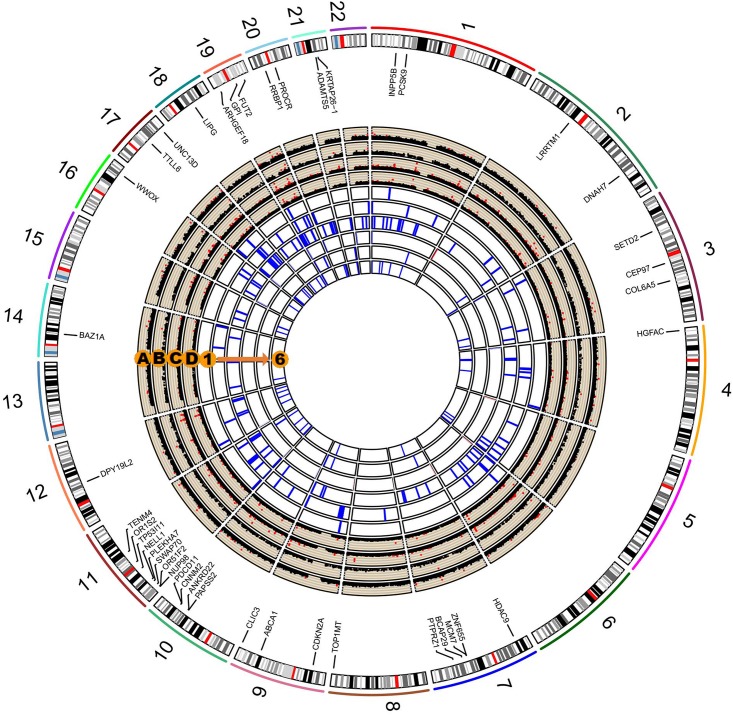
Circos plot integrating the results of single-variant, gene-based, and pathway-based analysis. The labeled genes are those significantly associated with ACS in both our study and in the existing *in silico* meta-GWAS dataset. Circos Manhattan plots of single-variant and gene-based analysis in our study participants (A and B); Circos Manhattan plots of single-variant and gene-based analysis in the meta-GWAS dataset (C and D). The six most significant pathways identified in our dataset and validated by the meta-GWAS dataset: (1) GO cyclin dependent protein serine threonine kinase inhibitor activity; (2) KEGG RIG-I-like receptor signaling pathway; (3) GO organophosphate ester transport; (4) GO negative regulation of muscle cell differentiation; (5) GO phospholipid efflux; (6) GO phospholipid transport.

We performed a series of gene-based tests aggregating deleterious missense or splicing variants with MAF < 0.05. Burden test is more powerful when a large proportion of variants are causal and effects are in the same direction, whereas SKAT test is designed to detect scenarios in which the effects of the aggregated variants have a different direction or magnitude. This testing regime identified four genes, *ANXA7*, *ZNF655*, *ZNF347*, and *ZNF750*, with exome-wide significant evidence for association (*P*_*gene*_ < 5.5 × 10^–6^, Table 3). Furthermore, we confirmed the association between *ZNF655* and disease risk using the meta-GWAS dataset (Table 3, *P* = 0.005). Considering that non-synonymous or splicing variants usually exert their biological function through influencing the expression of the host genes, we conducted differential expression analysis using GSE66360. As shown in Supplementary Figure S6, *ZNF655* was significantly downregulated in MI cases (*P* = 2.4 × 10^–9^). KEGG enrichment analysis showed that co-expressed genes of *ZNF655* were significantly enriched in protein processing in the endoplasmic reticulum, spliceosome pathway, and some disease pathways, including Huntington’s, Parkinson’s and Alzheimer’s disease (Supplementary Table S6). Additionally, genes significantly associated (*P* < 0.05) with ACS in both the discovery and replication datasets are provided in Figure 2.

**TABLE 3:** The results of gene-based analysis and validation using the *in silico* meta-GWAS dataset.

Gene	Chr.	Discovery analysis			Replication analysis	
			
		SNVs	Burden *P*-value	SKAT *P*-value	SNVs	*P*-value
*ANXA7*	10	4	1.06 × 10^–15^	8.23 × 10^–16^	2	0.200
*ZNF655*	7	6	1.83 × 10^−10^	4.22 × 10^−11^	4	0.005
*ZNF347*	19	3	5.73 × 10^–8^	3.93 × 10^–8^	3	0.684
*ZNF750*	17	2	7.87 × 10^–8^	1.15 × 10^–7^	5	0.075

### Pathway-Based Analysis

We further performed pathway-based analysis to examine the joint effect of genes within the same pathway. Although no significant pathway has been identified to be associated with ACS after Bonferroni correction, we summarized the 32 candidate disease-associated pathways, which demonstrated nominal association with ACS in both our study samples and the meta-GWAS dataset with a *P*-value less than 0.05 (Supplementary Table S7). The six top-ranking pathways were cyclin-dependent protein serine/threonine kinase inhibitor activity, RIG-I-like receptor signaling pathway, organophosphate ester transport, negative regulation of muscle cell differentiation, phospholipid efflux, and phospholipid transport (Figure 2). Several previously reported pathways were also replicated in our study, including low-density lipoprotein particle receptor binding, positive regulation of triglyceride metabolic process, reverse cholesterol transport, and lipoprotein metabolic pathways (Makinen et al., 2014; Ghosh et al., 2015; Zhao et al., 2016).

## Discussion

In this study, we used a novel control selection procedure and performed an EWAS of ACS using 1,669 cases and 1,935 controls from the Chinese population. We identified one promising protein-altering variation, rs10409124 at *STRN4*, which showed potential “universal” impact on ACS in both the European and East Asian populations. Additionally, gene-based analysis also provided several candidate genes, including *ANXA7*, *ZNF655*, *ZNF347*, and *ZNF750*, which demonstrated gene-wide significant association with ACS, and further confirmed one of them (*ZNF655*) in European samples. Moreover, gene-set enrichment analysis also provided several crucial pathways, such as cyclin-dependent protein, RIG-I-like receptor signaling, and phospholipid related pathways, which showed possible functional relevance in the pathogenesis of ACS. To our knowledge, this is the first genetic research combining an EWAS and risk prediction model to investigate the impact of single genetic variants and their aggregate effect on ACS risk.

Previous studies and our data indicated that some variants discovered in the European ancestry populations showed a weak or no association with CAD in other ethnic groups (Wang et al., 2011; Lu et al., 2012). Therefore, it is necessary to conduct genetic association studies in non-European populations to discover additional genetic risk factors. Considering that allele frequencies and LD patterns vary with ancestry, some genetic risk loci discovered in East Asians could not be successfully replicated in European (Wang et al., 2011; Lu et al., 2012; van der Harst and Verweij, 2018). For instance, Wang et al. (2011) identified a SNP, rs6903956, in *C6orf105* associated with susceptibility to CAD in the Chinese population. However, this SNP has not yet been confirmed as a susceptibility locus in European populations. Therefore, the failure of our study to replicate any promising variants in the *in silico* datasets conducted in Europeans could be understandable. Although non-significant, this exome-wide study revealed several interesting signals that can become a useful complement to CAD genetic susceptibility loci among the Chinese population. The most promising marker identified in this study was rs10409124 at *STRN4*. This variant reached exome-wide significant threshold in our study population and further showed nominal association with MI in European samples. The rs10409124 is located on the 13th exon of *STRN4*, resulting in a substitution of valine by isoleucine at site 568. *STRN4* maps at 19q13.2 and is involved in protein domain-specific binding and calmodulin binding (Lin et al., 2017). *STRN4* belongs to the striatin family of scaffold proteins that are highly expressed in the nervous system and are also known to form complexes with protein phosphatases and protein kinases (Wong et al., 2014).

We observed a gene-wide significant association between *ZNF655* and the risk of ACS in two gene-based tests including six high-impact low-frequency variants (*P*_*burden*_ = 1.83 × 10^–10^; *P*_*SKAT*_ = 4.22 × 10^–11^). This association was further confirmed by gene-based analysis in European samples. *ZNF655* locates at 7q22.1 and is overexpressed in adipocyte, heart, and B-lymphocytes. It encodes the Vav-interacting Krüppel-like factor 1, which is involved in DNA binding and protein-protein interactions (Houlard et al., 2005). Vik-1 belongs to the Krüppel-like factors (KLFs) protein family, which regulate the metabolic pathways across various tissues. KLFs have been shown to interact with the components of atherosclerosis pathogenesis and have also been linked to metabolic abnormalities, including obesity and diabetes mellitus (Pollak et al., 2018). In line with our findings from KEGG enrichment analysis of co-expressed genes of *ZNF655*, Zhao et al. (2016) constructed a genetic network based on gene-gene interactions and revealed that pathways involved in Alzheimer’s disease, non-alcoholic fatty liver disease, and Huntington’s disease were also associated with CAD risk. Moreover, Bis et al. (2018) recently identified the importance of *ZNF655* for transcriptional regulation in Alzheimer’s disease pathogenesis through whole genome sequencing. These findings illustrated that CAD and neurological disorders may share common pathogenic pathways. Further experimental studies are needed to explore the underlying biological mechanisms behind these statistical associations.

In the pathway-based analysis, 32 candidate pathways demonstrated potential relevance in the pathogenesis of ACS, although none of them passed the Bonferroni correction. The identified pathways reflected several different biological processes (such as cellular response and cell cycling), biological systems (such as the immune and endocrine systems), and signaling pathways perturbed by key genes (such as *TLR3*, *NFKB1*, and *PYK2*). In addition, the results also confirmed the association of some previously known processes, e.g., lipid metabolism. Ghosh et al. (2015) carried out a pathway enrichment analysis integrating several CAD-GWAS datasets. In accordance with our findings, they reported the associated pathways relevant to cellular integrity and CAD. Our findings are also consistent with the study conducted by Nair et al. (2014), which identified the importance of the regulation of nuclear factor kappa B1 in the development of CAD. Moreover, previous experimental studies (Nair et al., 2014) have already established the crucial role of the NFκB family in regulating many processes of significance to the disease state of the cardiovascular system including inflammation, cell proliferation, ischemia, etc.

The selection of controls is always a challenge in any genetic study. Absence of the disease of interest at enrollment is a common definition of control samples in many GWAS. However, one potential pitfall is that these controls might be latent cases, developing the disease of interest later in life. This confounding situation is worse when studying common diseases and results in loss of statistical power in studies and biased results (Manchia et al., 2013; Smith et al., 2013). There is always a trade-off between phenotypic refinement and study sample size. Manchia et al. (2013) expressed the view that accuracy of phenotypes is more important than a large sample size in detecting genetic associations. To overcome this limitation, previous studies have used the strategy of choosing disease-free participants at an older age as their control samples when studying aging-related diseases (Mitchell et al., 2014). Nevertheless, making comparisons between young cases and aged controls may introduce bias stemming from gaps in longevity and mismatching for potential covariates (Luo et al., 2007). In contrast, we proposed to employ a well-developed risk prediction tool to evaluate every subject’s disease risk and then selecting lower risk samples as controls. This strategy should be an efficient method to minimize misclassification rates in the era of electronic health records (EHR). This is also a cost-effective way to select “truly disease-free” controls instead of using large-scale invasive screening when utilizing the existing large number of publicly available controls.

This EWAS included 1,669 well-defined hospital-based ACS cases and 1,935 controls with low or moderate risk of 10-year ASCVD predicted using the China-PAR equation. Additionally, the genotype data from *in silico* dataset was used to provide supporting evidence, which further confirmed the reliability and reproducibility of our results. However, several limitations of this study are apparent and need to be addressed. Firstly, coverage of rare variants on the exome-array was suboptimal among the Chinese study population and this might limit the effective statistical power. Exome/whole-genome sequencing is needed to warrant the coverage of population-specific rare variants. Secondly, the sample size of the discovery cohort was relatively small resulting in a lack of sufficient statistical power to detect low frequency or rare variants with modest effect size. Further studies with larger-scale samples will be needed to replicate these promising findings. Thirdly, CAD is not a single disease but a collective term for a set of heterogeneous diseases with different but frequently overlapping pathogeneses. Clinically, the presentation of atherosclerotic CAD ranges from completely asymptomatic (subclinical atherosclerosis), angina pectoris (typical or atypical, stable or unstable), and silent MI to acute myocardial infarction (AMI) or sudden cardiac death. Potential phenotypic heterogeneity may exist between ACS and CAD; hence, the results need to be interpreted with caution. However, Nelson et al. (2017) found that there was strong concordance between corresponding genetic signals for the soft and hard definitions of CAD. In addition, we did not find significant genetic heterogeneity between the different disease subtypes. Next, our study used existing replication samples derived from European subjects. Considering the difference in MAF between geographic populations, lack of the independent replication samples from the Chinese population might lead to the failure of replication for some causal variants. Nevertheless, consistent signals between ethnicities were more likely to be valid findings. Lastly, this study mainly focused on providing statistical associations between genetic variants and ACS risk; however, the biological mechanisms underlying these associations still remain unclear.

## Conclusion

In summary, the current study reported an exome-chip association analysis of ACS, integrating a risk prediction model into control selection, thereby lowering the misclassification bias and increasing statistical power. By genotyping 1,669 cases and 1,935 controls and performing *in silico* replication, non-significant variants were identified except for one gene, *ZNF655*. Further gene-set enrichment analysis also provided some indications relevant to the pathogenesis of ACS. Future studies with larger sample size and refined phenotypes are needed to validate the promising associations. Our findings highlighted the importance of conducting genetic association studies in different ethnic populations.

## Data Availability Statement

Summary statistics of the discovery-stage is available from the Supplementary Material (Supplementary Data Sheet S1). Other datasets analyzed during this study were derived from the following public domain resources: Summary statistics of the replication stage is available from CARDIoGRAMplusC4D Consortium (http://www.cardiogramplusc4d.org/data-downloads/) and gene expression datasets is available from GEO Profiles (https://www.ncbi.nlm.nih.gov/geo/query/acc.cgi?acc=GSE66360).

## Ethics Statement

This study was approved by the Medical Ethics Committee of Peking University First Hospital. The patients/participants provided their written informed consent to participate in this study.

## Author Contributions

DC and YH conceived the study, undertook project leadership, and are guarantors of this work. QZ wrote the first draft of the manuscript. QZ and DC analyzed and interpreted the data. QZ, YZ, JieJ, JiaJ, FF, YG, ZW, QS, DC, and YH contributed to the drafting and critical revision of the manuscript. YZ, JieJ, FF, YG, ZW, and QS were involved in the sample collection, selection, and phenotype data preparation for the ACS genetic study and PUMMA study. YZ, JieJ, and JiaJ were involved in the database management for the ACS genetic and PUMMA cohort. All authors read and approved the final version of the manuscript.

## Conflict of Interest

The authors declare that the research was conducted in the absence of any commercial or financial relationships that could be construed as a potential conflict of interest.

## Footnotes

1 http://software.broadinstitute.org/gsea/msigdb

## Abbreviations

ACS: acute coronary syndrome
CAD: coronary artery disease
CVD: cardiovascular disease
EWAS: exome-wide association study
GWAS: genome-wide association study
LD: linkage disequilibrium
MAF: minor allele frequency
MI: myocardial infarction
NSTEMI: non-ST-segment elevation myocardial infarction
OR: odds ratio
SNP: single nucleotide polymorphism
STEMI: ST-segment elevation myocardial infarction
UA: unstable angina.
