# Supplementary material for: Exome-Wide Association Study Reveals Several Susceptibility Genes and Pathways Associated With Acute Coronary Syndromes in Han Chinese
Source: Front Genet. 2020 Apr 9;11:336. doi: 10.3389/fgene.2020.00336 (PMC7160370; doi:10.3389/fgene.2020.00336)

**SUPPLEMENTARY MATERIAL**

**Supplementary Table S1.** Baseline characteristics of the 4457 candidate controls in PUUMA cohorts, by 10-Year ASCVD risk.

|  | **Total (N=4457)** | **Low-risk (N=1698)** | **Moderate-risk (N=1347)** | **High-risk (N=1412)** | ***P*** |
| --- | --- | --- | --- | --- | --- |
| Age, mean(sd), y | 56.24 (8.60) | 50.11 (4.89) | 55.98 (5.56) | 63.86 (8.49) | <0.001 |
| Gender, female.(%) | 2808 (63.0) | 1417 (83.5) | 790 (58.6) | 601 (42.6) | <0.001 |
| SBP, mean(sd), mmHg | 133.28 (16.43) | 122.22 (11.19) | 134.44 (12.62) | 145.48 (15.89) | <0.001 |
| DBP, mean(sd), mmHg | 75.27 (9.81) | 72.34 (8.08) | 77.12 (9.29) | 77.02 (11.24) | <0.001 |
| TC, mean(sd), mg/dL | 206.35 (38.17) | 203.30 (34.84) | 209.40 (38.84) | 207.11 (41.02) | <0.001 |
| HDL-C, mean(sd), mg/dL | 55.59 (14.66) | 60.00 (14.98) | 54.23 (13.24) | 51.57 (14.15) | <0.001 |
| WC, mean(sd), cm | 82.75 (8.52) | 78.40 (7.76) | 84.19 (7.77) | 86.62 (7.70) | <0.001 |
| Current smoker, Yes. (%) | 897 (20.1) | 163 (9.6) | 330 (24.5) | 404 (28.6) | <0.001 |
| Diabetes, Yes. (%) | 990 (22.2) | 79 (4.7) | 223 (16.6) | 688 (48.7) | <0.001 |
| Family history of ASCVD, Yes. (%) | 998 (22.4) | 437 (25.7) | 322 (23.9) | 239 (16.9) | <0.001 |
| Antihypertensive treatment within 2 weeks, Yes. (%) | 1203 (27.0) | 111 ( 6.5) | 374 (27.8) | 718 (50.8) | <0.001 |

**Supplementary Table S2.** Summary of association results for 66 previously identified coronary artery disease loci.

| **Locus** | **Published variant** | | | | | | **Our study** | | | | |
| --- | --- | --- | --- | --- | --- | --- | --- | --- | --- | --- | --- |
|  | **Marker name** | **Chr:Pos (hg19)** | **EA/**  **OA** | **EAF** | **OR (95% CI)** | ***P* value** | **Lead SNP/**  **Proxy SNP** | **EAF** | **OR (95% CI)** | ***P* value** | **Matching direction of effect** |
| PCSK9 | rs11206510 | 1:55496039 | T/C | 0.834 | 1.06 (1.04,1.09) | 3.70E-08 | rs11206510 | 0.945 | 1.13 (0.88-1.46) | 3.26E-01 | Y |
| PPAP2B | rs17114036 | 1:56962821 | A/G | 0.910 | 1.08 (1.05,1.12) | 4.80E-08 | rs17114036 | 0.973 | 1.10 (0.78-1.57) | 5.85E-01 | Y |
| SORT1 | rs646776 | 1:109818530 | T/C | 0.780 | 1.12 (1.09,1.14) | 1.10E-23 | rs599839* | 0.931 | 1.23 (0.97-1.54) | 8.62E-02 | Y |
| IL6R | rs4845625 | 1:154422067 | T/C | 0.426 | 1.04 (1.02,1.06) | 1.10E-05 | rs4845625 | 0.489 | 0.95 (0.84-1.06) | 3.47E-01 | N |
| MIA3 | rs17464857 | 1:222762709 | T/G | 0.856 | 1.06 (1.04,1.09) | 6.90E-07 | rs17464857 | 0.997 | 0.97 (0.37-2.53) | 9.49E-01 | N |
|  | rs17465637 | 1:222823529 | C/A | 0.685 | 1.07 (1.05,1.09) | 1.60E-13 | rs17465637 | 0.603 | 1.20 (1.07-1.35) | 2.78E-03 | Y^#^ |
| AK097927 | rs16986953 | 2:19942473 | A/G | 0.073 | 1.11 (1.08,1.15) | 4.80E-10 | - | - | - | - | - |
| APOB | rs515135 | 2:21286057 | C/T | 0.817 | 1.05 (1.03,1.07) | 3.70E-06 | rs515135 | 0.888 | 1.14 (0.95-1.38) | 1.51E-01 | Y |
| ABCG5/ABCG8 | rs6544713 | 2:44073881 | T/C | 0.319 | 1.06 (1.04,1.08) | 3.70E-09 | rs6544713 | 0.006 | 1.16 (0.58-2.34) | 6.72E-01 | Y |
| VAMP5/VAMP8/GGCX | rs1561198 | 2:85809989 | T/C | 0.464 | 1.06 (1.04,1.07) | 4.00E-11 | rs1561198 | 0.388 | 1.11 (0.99-1.25) | 8.48E-02 | Y |
| ZEB2/AC074093.1 | rs2252641 | 2:145801461 | C/T | 0.467 | 1.03 (1.01,1.05) | 6.20E-04 | rs2252641 | 0.763 | 0.89 (0.77-1.02) | 8.03E-02 | N |
| WDR12 | rs6725887 | 2:203745885 | C/T | 0.116 | 1.12 (1.09,1.15) | 7.80E-19 | - | - | - | - | - |
| KCNJ13/GIGYF2 | rs1801251 | 2:233633460 | A/G | 0.364 | 1.04 (1.02,1.06) | 5.20E-06 | rs1801251 | 0.301 | 1.01 (0.89-1.15) | 8.70E-01 | Y |
| MRAS | rs9818870 | 3:138122122 | T/C | 0.144 | 1.07 (1.05,1.09) | 7.80E-09 | rs9818870 | 0.007 | 0.82 (0.39-1.73) | 6.08E-01 | N |
| REST/NOA1 | rs17087335 | 4:57838583 | T/G | 0.203 | 1.05 (1.03,1.08) | 8.40E-08 | rs17081935* | 0.374 | 1.11 (0.98-1.25) | 9.20E-02 | Y |
| EDNRA | rs1878406 | 4:148393664 | T/C | 0.150 | 1.07 (1.04,1.09) | 3.90E-09 | - | - | - | - | - |
| GUCY1A3 | rs7692387 | 4:156635309 | G/A | 0.814 | 1.04 (1.02,1.06) | 4.30E-04 | rs7692387 | 0.817 | 0.96 (0.83-1.11) | 5.64E-01 | N |
| SLC22A4/SLC22A5 | rs273909 | 5:131667353 | G/A | 0.116 | 1.05 (1.02,1.09) | 9.90E-04 | - | - | - | - | - |
| ADTRP/C6orf105 | rs6903956 | 6:11774583 | G/A | 0.636 | 1.00 (0.99,1.02) | 7.20E-01 | - | - | - | - | - |
| PHACTR1 | rs12526453 | 6:12927544 | C/G | 0.693 | 1.09 (1.08,1.11) | 1.60E-23 | - | - | - | - | - |
| C2 | rs3130683 | 6:31888367 | T/C | 0.860 | 1.08 (1.05,1.11) | 2.80E-08 | rs3130683 | 0.978 | 1.26 (0.86-1.85) | 2.35E-01 | Y |
| ANKS1A | rs17609940 | 6:35034800 | G/C | 0.808 | 1.03 (1.01,1.05) | 7.10E-03 | rs12205331* | 0.968 | 1.08 (0.78-1.48) | 6.59E-01 | Y |
| KCNK5 | rs10947789 | 6:39174922 | T/C | 0.765 | 1.04 (1.02,1.06) | 1.90E-04 | rs10947789 | 0.793 | 1.11 (0.96-1.28) | 1.58E-01 | Y |
| TCF21 | rs12190287 | 6:134214525 | C/G | 0.622 | 1.06 (1.03,1.08) | 2.30E-06 | - | - | - | - | - |
| SLC22A3/LPAL2/LPA/PLG | rs2048327 | 6:160863532 | C/T | 0.366 | 1.06 (1.04,1.08) | 7.40E-12 | rs2048327 | 0.431 | 0.94 (0.84-1.06) | 3.05E-01 | N |
|  | rs3798220 | 6:160961137 | C/T | 0.019 | 1.49 (1.40,1.59) | 9.40E-33 | rs3798220 | 0.082 | 0.80 (0.65-0.99) | 3.98E-02 | N |
|  | rs4252120 | 6:161143608 | T/C | 0.729 | 1.04 (1.02,1.06) | 8.20E-05 | rs4252120 | 0.997 | 0.71 (0.28-1.80) | 4.73E-01 | N |
| HDAC9 | rs2023938 | 7:19036775 | C/T | 0.101 | 1.08 (1.05,1.11) | 1.70E-08 | rs2023938 | 0.002 | 3.59 (1.05-12.3) | 4.23E-02 | Y^#^ |
| 7q22 | rs10953541 | 7:107244545 | C/T | 0.757 | 1.03 (1.01,1.05) | 5.80E-03 | rs10953541 | 0.844 | 1.01 (0.87-1.19) | 8.73E-01 | Y |
| ZC3HC1 | rs11556924 | 7:129663496 | C/T | 0.657 | 1.07 (1.05,1.09) | 6.30E-13 | rs11556924 | 0.953 | 1.08 (0.82-1.43) | 5.78E-01 | Y |
| NOS3 | rs3918226 | 7:150690176 | T/C | 0.071 | 1.13 (1.09,1.17) | 1.60E-12 | - | - | - | - | - |
| LPL | rs264 | 8:19813180 | G/A | 0.859 | 1.06 (1.04,1.09) | 9.80E-07 | rs264 | 0.805 | 0.96 (0.83-1.11) | 5.76E-01 | N |
| TRIB1 | rs2954029 | 8:126490972 | A/T | 0.541 | 1.06 (1.05,1.08) | 5.20E-13 | rs2954029 | 0.410 | 1.11 (0.98-1.25) | 9.07E-02 | Y |
| CDKN2BAS | rs3217992 | 9:22003223 | T/C | 0.380 | 1.13 (1.11,1.15) | 5.50E-49 | rs3217992 | 0.488 | 1.08 (0.96-1.21) | 2.19E-01 | Y |
|  | rs4977574 | 9:22098574 | G/A | 0.486 | 1.19 (1.17,1.21) | 4.70E-101 | rs4977574 | 0.478 | 1.18 (1.05-1.33) | 5.50E-03 | Y |
| SVEP1 | rs111245230 | 9:113169775 | C/T | 0.036 | 1.12 (1.07,1.17) | 8.30E-07 | rs111245230 | 0.000 | 5.85 (0.31-109.4) | 2.37E-01 | Y |
| ABO | rs579459 | 9:136154168 | C/T | 0.215 | 1.07 (1.05,1.09) | 5.80E-11 | rs579459 | 0.216 | 1.07 (0.93-1.23) | 3.39E-01 | Y |
| KIAA1462 | rs2505083 | 10:30335122 | C/T | 0.411 | 1.06 (1.04,1.08) | 5.50E-12 | rs2505083 | 0.237 | 1.12 (0.98-1.28) | 9.79E-02 | Y |
| CXCL12 | rs2047009 | 10:44539913 | G/T | 0.494 | 1.05 (1.03,1.07) | 1.20E-09 | - | - | - | - | - |
|  | rs501120 | 10:44753867 | T/C | 0.834 | 1.07 (1.05,1.09) | 6.30E-11 | rs501120 | 0.620 | 0.98 (0.87-1.10) | 6.80E-01 | N |
| LIPA | rs11203042 | 10:90989109 | T/C | 0.446 | 1.04 (1.02,1.06) | 3.90E-06 | rs11203042 | 0.532 | 0.96 (0.86-1.09) | 5.49E-01 | N |
|  | rs1412444 | 10:91002927 | T/C | 0.358 | 1.07 (1.05,1.08) | 8.20E-14 | - | - | - | - | - |
| CYP17A1/CNNM2/NT5C2 | rs12413409 | 10:104719096 | G/A | 0.902 | 1.07 (1.04,1.10) | 1.70E-07 | rs12413409 | 0.736 | 1.18 (1.04-1.35) | 1.07E-02 | Y |
| SWAP70 | rs10840293 | 11:9751196 | A/G | 0.552 | 1.05 (1.03,1.07) | 6.90E-09 | rs415895* | 0.452 | 1.05 (0.93-1.18) | 4.35E-01 | Y |
| MRVI1/CTR9 | rs11042937 | 11:10745394 | T/G | 0.511 | 1.01 (0.99,1.03) | 2.00E-01 | rs11042937 | 0.835 | 1.24 (1.06-1.44) | 8.33E-03 | Y^#^ |
| PDGFD | rs974819 | 11:103660567 | T/C | 0.316 | 1.06 (1.04,1.08) | 1.80E-10 | rs974819 | 0.627 | 1.11 (0.98-1.25) | 9.55E-02 | Y |
| ZNF259/APOA5/APOA1 | rs964184 | 11:116648917 | G/C | 0.163 | 1.05 (1.03,1.08) | 4.70E-06 | rs964184 | 0.216 | 1.15 (1.00-1.33) | 4.75E-02 | Y |
| LRP1 | rs11172113 | 12:57527283 | C/T | 0.408 | 1.04 (1.02,1.05) | 2.40E-05 | rs11172113 | 0.235 | 1.04 (0.91-1.20) | 5.40E-01 | Y |
| ATP2B1 | rs7136259 | 12:90081188 | T/C | 0.426 | 1.03 (1.02,1.05) | 5.60E-05 | - | - | - | - | - |
| SH2B3 | rs3184504 | 12:111884608 | T/C | 0.442 | 1.06 (1.04,1.08) | 7.50E-11 | rs3184504 | 0.005 | 1.70 (0.78-3.70) | 1.85E-01 | Y |
| KSR2 | rs11830157 | 12:118265441 | G/T | 0.382 | 1.03 (1.01,1.04) | 1.70E-03 | - | - | - | - | - |
| SCARB1 | rs11057830 | 12:125307053 | A/G | 0.147 | 1.07 (1.05,1.10) | 4.20E-09 | rs11057830 | 0.137 | 0.91 (0.76-1.07) | 2.43E-01 | N |
| FLT1 | rs9319428 | 13:28973621 | A/G | 0.306 | 1.04 (1.03,1.06) | 1.00E-06 | rs9319428 | 0.413 | 1.06 (0.94-1.20) | 3.33E-01 | Y |
| COL4A1/COL4A2 | rs4773144 | 13:110960712 | G/A | 0.437 | 1.04 (1.02,1.06) | 2.80E-05 | - | - | - | - | - |
|  | rs9515203 | 13:111049623 | T/C | 0.752 | 1.06 (1.04,1.08) | 6.50E-10 | - | - | - | - | - |
| HHIPL1 | rs2895811 | 14:100133942 | C/T | 0.422 | 1.04 (1.03,1.06) | 9.10E-07 | rs2895811 | 0.276 | 1.04 (0.91-1.18) | 6.09E-01 | Y |
| SMAD3 | rs56062135 | 15:67455630 | C/T | 0.783 | 1.07 (1.05,1.09) | 6.40E-12 | rs17293632* | 0.979 | 1.19 (0.79-1.80) | 4.13E-01 | Y |
| ADAMTS7 | rs7173743 | 15:79141784 | T/C | 0.540 | 1.06 (1.04,1.08) | 6.40E-08 | rs7173743 | 0.575 | 1.07 (0.95-1.20) | 2.65E-01 | Y |
| MFGE8/ABHD2 | rs8042271 | 15:89574218 | G/A | 0.928 | 1.09 (1.05,1.12) | 6.10E-07 | - | - | - | - | - |
| FURIN/FES | rs17514846 | 15:91416550 | A/C | 0.469 | 1.06 (1.04,1.08) | 1.30E-10 | rs17514846 | 0.157 | 1.20 (1.03-1.41) | 2.25E-02 | Y |
| CETP | rs1800775 | 16:56995236 | C/A | 0.511 | 1.04 (1.02,1.06) | 2.50E-06 | rs1800775 | 0.450 | 1.01 (0.90-1.13) | 9.01E-01 | Y |
| SMG6 | rs216172 | 17:2126504 | C/G | 0.356 | 1.04 (1.02,1.05) | 2.30E-05 | rs216172 | 0.265 | 1.03 (0.91-1.18) | 6.24E-01 | Y |
| RAI1/PEMT/RASD1 | rs12936587 | 17:17543722 | G/A | 0.540 | 1.03 (1.01,1.04) | 1.80E-03 | rs12936587 | 0.889 | 1.10 (0.92-1.32) | 3.08E-01 | Y |
| UBE2Z | rs46522 | 17:46988597 | T/C | 0.527 | 1.03 (1.02,1.05) | 9.30E-05 | rs46522 | 0.713 | 1.14 (1.00-1.29) | 4.62E-02 | Y |
| BCAS3 | rs7212798 | 17:59013488 | C/T | 0.150 | 1.07 (1.05,1.10) | 2.20E-08 | rs8080784* | 0.127 | 1.19 (0.99-1.41) | 5.27E-02 | Y |
| PMAIP1/MC4R | rs663129 | 18:57838401 | A/G | 0.248 | 1.04 (1.02,1.06) | 1.80E-05 | rs571312* | 0.218 | 1.17 (1.02-1.35) | 2.34E-02 | Y |
| ANGPTL4 | rs116843064 | 19:8429323 | G/A | 0.980 | 1.17 (1.10,1.25) | 2.90E-07 | - | - | - | - | - |
| LDLR | rs1122608 | 19:11163601 | G/T | 0.752 | 1.06 (1.04,1.08) | 3.70E-10 | rs1122608 | 0.901 | 1.17 (0.96-1.42) | 1.14E-01 | Y |
| ZNF507/LOC400684 | rs12976411 | 19:32882020 | A/T | 0.939 | 1.04 (1.00,1.07) | 2.90E-02 | - | - | - | - | - |
| APOE/APOC1 | rs2075650 | 19:45395619 | G/A | 0.133 | 1.08 (1.05,1.10) | 2.10E-09 | rs2075650 | 0.094 | 1.04 (0.85-1.27) | 6.88E-01 | Y |
|  | rs445925 | 19:45415640 | G/A | 0.891 | 1.10 (1.07,1.13) | 1.20E-12 | rs445925 | 0.922 | 1.29 (1.03-1.61) | 2.68E-02 | Y |
| Gene desert/KCNE2 | rs9982601 | 21:35599128 | T/C | 0.134 | 1.10 (1.07,1.12) | 1.70E-14 | rs9982601 | 0.002 | 0.50 (0.12-1.99) | 3.24E-01 | N |
| POM121L9P/ADORA2A | rs180803 | 22:24658858 | G/T | 0.977 | 1.18 (1.12,1.24) | 7.10E-10 | - | - | - | - | - |

*We report proxy markers in high linkage disequilibrium with the GWAS variants at 7 loci: SORT1 (rs646776 and rs599839; r^2^=0.87), REST/NOA1 (rs17087335 and rs17081935; r^2^=0.98), ANKS1A (rs17609940 and rs12205331; r^2^=1.0), SWAP70 (rs10840293 and rs415895; r^2^=0.84), SMAD3 (rs56062135 and rs17293632, r^2^=1.0), BCAS3 (rs7212798 and rs8080784; r^2^=1.0), PMAIP1/MC4R (rs663129 and rs571312; r^2^=1.0). The LD metric r^2^ was estimated in the 1000 Genome CHB population.

^#^ indicated nominal statistically significance between two estimates (previous GWAS and our study populations) among 42 consistent direction SNPs.

**Supplementary Table S3.** The results of variants that were found to be potentially associated with acute coronary syndromes at the discovery stage.

| **Variant ID** | **Chr.** | **Pos.** | **Major/Minor allele** | **Genotype** | **MAF** | **Variant** | **Gene** | **Type** | **OR(95%CI)** | ***P* ^a^** | ***P* ^b^** |
| --- | --- | --- | --- | --- | --- | --- | --- | --- | --- | --- | --- |
| rs117506953 | 19 | 52825095 | C/T | 0/317/3219 | 0.045 | Leu121Phe | ZNF480 | nonsynonymous | 10.6(6.87-16.5) | 6.0E-26 | 3.9E-31 |
| rs10409124 | 19 | 47226434 | C/T | 3/497/3065 | 0.071 | Val568Ile | STRN4 | nonsynonymous | 3.87(2.97-5.03) | 6.6E-24 | 2.3E-24 |
| rs73929373 | 19 | 34706531 | A/G | 0/111/3409 | 0.016 | Asn249Ser | LSM14A | nonsynonymous | 0.05(0.02-0.14) | 5.4E-10 | 7.4E-13 |
| rs4127353 | 11 | 58125620 | C/T | 151/1290/2095 | 0.225 | Tyr308Cys | OR5B17 | nonsynonymous | 1.45(1.25-1.67) | 3.1E-07 | 3.4E-07 |
| rs149822831 | 7 | 99170144 | T/C | 2/70/3486 | 0.010 | Phe138Ser | ZNF655 | nonsynonymous | 0.04(0.01-0.15) | 7.3E-07 | 4.3E-09 |
| rs182592231 | 10 | 75156333 | G/A | 3/89/3447 | 0.013 | Pro127Ser | ANXA7 | nonsynonymous | 0.01(0.00-0.09) | 1.7E-06 | 3.6E-13 |
| rs149721746 | 11 | 123810502 | A/G | 3/70/3459 | 0.011 | Tyr60Cys | OR4D5 | nonsynonymous | 0.12(0.05-0.29) | 2.2E-06 | 5.2E-07 |
| rs144650170 | 3 | 151535235 | A/G | 2/65/3459 | 0.010 | Thr74Ala | AADAC | nonsynonymous | 0.04(0.01-0.17) | 2.3E-06 | 3.6E-08 |
| rs189450639 | 7 | 48315825 | C/T | 0/54/3529 | 0.008 | His2188Tyr | ABCA13 | nonsynonymous | 0.07(0.02-0.22) | 6.1E-06 | 3.6E-07 |
| rs140441570 | 1 | 1431163 | G/A | 0/78/3445 | 0.011 | Arg638Gln | ATAD3B | nonsynonymous | 0.01(0.00-0.11) | 6.5E-06 | 1.1E-10 |
| rs4646422 | 15 | 75015305 | C/T | 71/984/2467 | 0.160 | Gly45Asp | CYP1A1 | nonsynonymous | 0.68(0.57-0.80) | 8.4E-06 | 8.6E-06 |
| rs185660005 | 16 | 84514305 | C/G | 47/39/3518 | 0.018 | Gly363Trp | TLDC1 | nonsynonymous | 2.38(1.62-3.49) | 9.6E-06 | 9.7E-06 |
| rs187565189 | 3 | 133302985 | C/T | 0/46/3529 | 0.006 | Pro153Thr | CDV3 | nonsynonymous | 0.05(0.01-0.19) | 9.6E-06 | 4.3E-07 |
| rs149276487 | 14 | 68249803 | A/C | 0/63/3490 | 0.009 | Cys1356Gly | ZFYVE26 | nonsynonymous | 0.15(0.06-0.35) | 1.3E-05 | 1.1E-05 |
| rs187217680 | 19 | 36159524 | G/A | 1/87/3463 | 0.013 | Ala85Thr | UPK1A | nonsynonymous | 0.21(0.10-0.43) | 2.5E-05 | 1.3E-05 |
| rs115046131 | 4 | 89671591 | A/C | 0/75/3485 | 0.011 | Ser642Ala | FAM13A | nonsynonymous | 0.08(0.02-0.26) | 3.1E-05 | 1.2E-06 |
| rs200612063 | 2 | 204305568 | A/G | 2/92/3476 | 0.013 | Leu782Pro | RAPH1 | nonsynonymous | 0.23(0.11-0.46) | 4.0E-05 | 2.7E-05 |
| rs189439132 | 12 | 57627816 | G/A | 94/99/3411 | 0.040 | Arg437His | SHMT2 | nonsynonymous | 1.69(1.30-2.18) | 5.8E-05 | 5.9E-05 |
| rs138391103 | 17 | 80789026 | G/C | 1/53/3489 | 0.008 | Asn435Lys | ZNF750 | nonsynonymous | 0.04(0.00-0.20) | 8.3E-05 | 1.6E-06 |

a. Derived from the logistic regression model after adjusting for age, gender and top two PCs as appropriate assuming an additive genetic model;

b. Derived from the Firth bias-corrected logistic likelihood ratio test after adjusting for age, gender and the top two principal components.

**Supplementary Table S4.** The results of promising variants at the replication stage.

| **VariantID** | **Chr.** | **Pos.** | **Major/Minor allele** | **MAF^a^** | **MAF^b^** | **Gene** | **OR(95%CI)** | ***P* value** |
| --- | --- | --- | --- | --- | --- | --- | --- | --- |
| rs117506953 | 19 | 52825095 | C/T | 0.045 | 0.003 | ZNF480 | 1.32(0.87-16.6) | 0.1807 |
| **rs10409124** | **19** | **47226434** | **C/T** | **0.071** | **0.003** | **STRN4** | **1.34(1.03-15.8)** | **0.02547** |
| rs73929373 | 19 | 34706531 | A/G | 0.016 | 0.040 | LSM14A | 1.07(0.93-8.73) | 0.314 |
| rs4127353 | 11 | 58125620 | C/T | 0.225 | 0.373 | OR5B17 | 1.01(0.99-7.36) | 0.1641 |
| rs149822831 | 7 | 99170144 | T/C | 0.010 |  | ZNF655 | - | - |
| rs182592231 | 10 | 75156333 | G/A | 0.013 |  | ANXA7 | - | - |
| rs149721746 | 11 | 123810502 | A/G | 0.011 | 0.001 | OR4D5 | 3.85(0.82-4221) | 0.08509 |
| rs144650170 | 3 | 151535235 | A/G | 0.010 |  | AADAC | - | - |
| rs189450639 | 7 | 48315825 | C/T | 0.008 |  | ABCA13 | - | - |
| rs140441570 | 1 | 1431163 | G/A | 0.011 | 0.018 | ATAD3B | 1.32(0.95-15.9) | 0.08925 |
| rs4646422 | 15 | 75015305 | C/T | 0.160 |  | CYP1A1 | - | - |
| rs185660005 | 16 | 84514305 | C/G | 0.018 |  | TLDC1 | - | - |
| rs187565189 | 3 | 133302985 | C/T | 0.006 |  | CDV3 | - | - |
| rs149276487 | 14 | 68249803 | A/C | 0.009 | 0.002 | ZFYVE26 | 1.46(0.12-60.5) | 0.7594 |
| rs187217680 | 19 | 36159524 | G/A | 0.013 | 0.001 | UPK1A | 1.31(0.69-18.1) | 0.4065 |
| rs115046131 | 4 | 89671591 | A/C | 0.011 | 0.001 | FAM13A | 1.36(0.42-26.6) | 0.6004 |
| rs200612063 | 2 | 204305568 | A/G | 0.013 |  | RAPH1 | - | - |
| rs189439132 | 12 | 57627816 | G/A | 0.040 |  | SHMT2 | - | - |
| rs138391103 | 17 | 80789026 | G/C | 0.008 |  | ZNF750 | - | - |

^a^ minor allele frequency in our study dataset; ^b^ minor allele frequency in *in silico* meta GWAS dataset.

**Supplementary Table S5.** Associations of rs10409124 with ACS, subgroup analysis.

| **Variables** | | **case/control** | **OR (95%CI)** | ***P**** |
| --- | --- | --- | --- | --- |
| age | < 60 | 776/1587 | 3.48 (2.47-4.89) | 0.723 |
|  | ≥ 60 | 893/348 | 3.87 (2.37-6.34) |  |
| gender | male | 1286/534 | 3.35 (2.33-4.82) | 0.391 |
|  | female | 383/1401 | 4.28 (2.80-6.54) |  |
| BMI | < 24 | 517/610 | 4.62 (2.85-7.47) | 0.510 |
|  | ≥ 24 | 783/1352 | 3.79 (2.72-5.29) |  |
| smoke | current/ever | 941/367 | 4.43 (2.77-7.08) | 0.493 |
|  | never | 728/1568 | 3.61 (2.55-5.11) |  |
| hypertension | no | 800/1298 | 3.92 (2.80-5.48) | 0.699 |
|  | yes | 869/637 | 4.39 (2.73-7.06) |  |
| type 2 diabetes | no | 1344/1756 | 3.77 (2.84-5.02) | 0.488 |
|  | yes | 325/179 | 5.12 (2.27-11.5) |  |

*test for heterogeneity.

**Supplementary Table S6.** KEGG enrichment analysis of genes co-expressed with ZNF655 in myocardial infarction patients from GSE66360.

| **Pathway ID** | **Description** | **GeneRatio** | **BgRatio** | ***P* value** | ***P* adjust** |
| --- | --- | --- | --- | --- | --- |
| hsa04141 | Protein processing in endoplasmic reticulum | 96/1982 | 165/7470 | 4.58E-18 | 1.44E-15 |
| hsa03040 | Spliceosome | 82/1982 | 134/7470 | 1.94E-17 | 3.04E-15 |
| hsa04932 | Non-alcoholic fatty liver disease (NAFLD) | 88/1982 | 149/7470 | 3.15E-17 | 3.29E-15 |
| hsa05016 | Huntington disease | 98/1982 | 193/7470 | 3.19E-13 | 2.51E-11 |
| hsa05012 | Parkinson disease | 76/1982 | 142/7470 | 5.31E-12 | 3.34E-10 |
| hsa03050 | Proteasome | 34/1982 | 45/7470 | 8.37E-12 | 4.38E-10 |
| hsa03010 | Ribosome | 79/1982 | 153/7470 | 2.24E-11 | 1.01E-09 |
| hsa00190 | Oxidative phosphorylation | 68/1982 | 133/7470 | 9.79E-10 | 3.79E-08 |
| hsa05010 | Alzheimer disease | 82/1982 | 171/7470 | 1.09E-09 | 3.79E-08 |
| hsa05170 | Human immunodeficiency virus 1 infection | 95/1982 | 212/7470 | 4.68E-09 | 1.40E-07 |
| hsa04120 | Ubiquitin mediated proteolysis | 68/1982 | 137/7470 | 4.89E-09 | 1.40E-07 |
| hsa05169 | Epstein-Barr virus infection | 90/1982 | 201/7470 | 1.26E-08 | 3.29E-07 |
| hsa05166 | Human T-cell leukemia virus 1 infection | 96/1982 | 219/7470 | 1.54E-08 | 3.72E-07 |
| hsa04714 | Thermogenesis | 99/1982 | 229/7470 | 2.18E-08 | 4.62E-07 |
| hsa04660 | T cell receptor signaling pathway | 53/1982 | 101/7470 | 2.21E-08 | 4.62E-07 |
| hsa04144 | Endocytosis | 104/1982 | 244/7470 | 2.36E-08 | 4.63E-07 |
| hsa04659 | Th17 cell differentiation | 55/1982 | 107/7470 | 3.09E-08 | 5.72E-07 |
| hsa03013 | RNA transport | 76/1982 | 171/7470 | 2.50E-07 | 4.35E-06 |
| hsa03060 | Protein export | 18/1982 | 23/7470 | 3.22E-07 | 5.32E-06 |
| hsa05163 | Human cytomegalovirus infection | 94/1982 | 225/7470 | 3.46E-07 | 5.44E-06 |
| hsa05161 | Hepatitis B | 65/1982 | 144/7470 | 9.42E-07 | 1.41E-05 |
| hsa04218 | Cellular senescence | 69/1982 | 160/7470 | 3.35E-06 | 4.77E-05 |
| hsa03018 | RNA degradation | 40/1982 | 79/7470 | 3.83E-06 | 5.23E-05 |
| hsa05167 | Kaposi sarcoma-associated herpesvirus infection | 76/1982 | 186/7470 | 1.21E-05 | 1.58E-04 |
| hsa05131 | Shigellosis | 32/1982 | 65/7470 | 7.16E-05 | 8.89E-04 |
| hsa04140 | Autophagy - animal | 54/1982 | 128/7470 | 7.86E-05 | 8.89E-04 |
| hsa05203 | Viral carcinogenesis | 78/1982 | 201/7470 | 7.89E-05 | 8.89E-04 |
| hsa04650 | Natural killer cell mediated cytotoxicity | 55/1982 | 131/7470 | 7.93E-05 | 8.89E-04 |
| hsa05130 | Pathogenic Escherichia coli infection | 28/1982 | 55/7470 | 9.50E-05 | 1.02E-03 |
| hsa04217 | Necroptosis | 65/1982 | 162/7470 | 9.75E-05 | 1.02E-03 |
| hsa04066 | HIF-1 signaling pathway | 44/1982 | 100/7470 | 1.11E-04 | 1.12E-03 |
| hsa04211 | Longevity regulating pathway | 40/1982 | 89/7470 | 1.28E-04 | 1.26E-03 |
| hsa04658 | Th1 and Th2 cell differentiation | 41/1982 | 92/7470 | 1.33E-04 | 1.27E-03 |
| hsa04621 | NOD-like receptor signaling pathway | 66/1982 | 168/7470 | 1.81E-04 | 1.67E-03 |
| hsa04068 | FoxO signaling pathway | 54/1982 | 132/7470 | 2.06E-04 | 1.80E-03 |
| hsa05162 | Measles | 54/1982 | 132/7470 | 2.06E-04 | 1.80E-03 |
| hsa04216 | Ferroptosis | 21/1982 | 40/7470 | 4.10E-04 | 3.41E-03 |
| hsa04142 | Lysosome | 50/1982 | 123/7470 | 4.18E-04 | 3.41E-03 |
| hsa05168 | Herpes simplex infection | 70/1982 | 185/7470 | 4.30E-04 | 3.41E-03 |
| hsa04370 | VEGF signaling pathway | 28/1982 | 59/7470 | 4.34E-04 | 3.41E-03 |
| hsa04137 | Mitophagy - animal | 30/1982 | 65/7470 | 4.99E-04 | 3.73E-03 |
| hsa04210 | Apoptosis | 54/1982 | 136/7470 | 4.99E-04 | 3.73E-03 |
| hsa04662 | B cell receptor signaling pathway | 32/1982 | 71/7470 | 5.54E-04 | 4.04E-03 |
| hsa00510 | N-Glycan biosynthesis | 24/1982 | 49/7470 | 6.19E-04 | 4.42E-03 |
| hsa05212 | Pancreatic cancer | 33/1982 | 75/7470 | 7.72E-04 | 5.39E-03 |
| hsa05220 | Chronic myeloid leukemia | 33/1982 | 76/7470 | 1.02E-03 | 6.98E-03 |
| hsa04919 | Thyroid hormone signaling pathway | 46/1982 | 116/7470 | 1.30E-03 | 8.70E-03 |
| hsa00020 | Citrate cycle (TCA cycle) | 16/1982 | 30/7470 | 1.60E-03 | 1.05E-02 |
| hsa04625 | C-type lectin receptor signaling pathway | 41/1982 | 104/7470 | 2.64E-03 | 1.69E-02 |
| hsa04723 | Retrograde endocannabinoid signaling | 55/1982 | 148/7470 | 2.71E-03 | 1.70E-02 |
| hsa05120 | Epithelial cell signaling in Helicobacter pylori infection | 29/1982 | 68/7470 | 2.80E-03 | 1.72E-02 |
| hsa04110 | Cell cycle | 47/1982 | 124/7470 | 3.42E-03 | 2.06E-02 |
| hsa04722 | Neurotrophin signaling pathway | 45/1982 | 119/7470 | 4.33E-03 | 2.54E-02 |
| hsa03015 | mRNA surveillance pathway | 36/1982 | 91/7470 | 4.37E-03 | 2.54E-02 |
| hsa01200 | Carbon metabolism | 44/1982 | 116/7470 | 4.45E-03 | 2.54E-02 |
| hsa04213 | Longevity regulating pathway - multiple species | 26/1982 | 62/7470 | 5.92E-03 | 3.32E-02 |
| hsa05231 | Choline metabolism in cancer | 38/1982 | 99/7470 | 6.28E-03 | 3.46E-02 |
| hsa04150 | mTOR signaling pathway | 54/1982 | 151/7470 | 7.33E-03 | 3.97E-02 |
| hsa05211 | Renal cell carcinoma | 28/1982 | 69/7470 | 7.51E-03 | 4.00E-02 |
| hsa05164 | Influenza A | 60/1982 | 171/7470 | 7.78E-03 | 4.07E-02 |

**Supplementary Table S7.** Thirty-two candidate pathways that exhibited significant association with acute coronary syndromes in the present study and in the existing *in silico* meta-GWAS dataset.

| **Database** | **Pathway** | **Description of the identified pathway** | ***P* ^a^** | ***P* ^b^** |
| --- | --- | --- | --- | --- |
| GO | GO CYCLIN DEPENDENT PROTEIN SERINE THREONINE KINASE INHIBITOR ACTIVITY | Stops, prevents or reduces the activity of a cyclin-dependent protein serine/threonine kinase. | 5.51E-03 | 5.39E-04 |
| KEGG | KEGG RIG I LIKE RECEPTOR SIGNALING PATHWAY | Specific families of pattern recognition receptors are responsible for detecting viral pathogens and generating innate immune responses. Non-self RNA appearing in a cell as a result of intracellular viral replication is recognized by a family of cytosolic RNA helicases termed RIG-I-like receptors (RLRs). The RLR proteins include RIG-I, MDA5, and LGP2 and are expressed in both immune and nonimmune cells. Upon recognition of viral nucleic acids, RLRs recruit specific intracellular adaptor proteins to initiate signaling pathways that lead to the synthesis of type I interferon and other inflammatory cytokines, which are important for eliminating viruses. | 6.07E-03 | 1.48E-02 |
| GO | GO ORGANOPHOSPHATE ESTER TRANSPORT | The directed movement of organophosphate esters into, out of or within a cell, or between cells, by means of some agent such as a transporter or pore. Organophosphate esters are small organic molecules containing phosphate ester bonds. | 6.10E-03 | 4.55E-04 |
| GO | GO NEGATIVE REGULATION OF MUSCLE CELL DIFFERENTIATION | Any process that stops, prevents, or reduces the frequency, rate or extent of muscle cell differentiation. | 7.91E-03 | 4.93E-02 |
| GO | GO PHOSPHOLIPID EFFLUX | The directed movement of a phospholipid out of a cell or organelle. | 8.64E-03 | 3.47E-02 |
| GO | GO PHOSPHOLIPID TRANSPORT | The directed movement of phospholipids into, out of or within a cell, or between cells, by means of some agent such as a transporter or pore. Phospholipids are any lipids containing phosphoric acid as a mono- or diester. | 9.68E-03 | 1.91E-04 |
| GO | GO CELLULAR RESPONSE TO VITAMIN D | Any process that results in a change in state or activity of a cell (in terms of movement, secretion, enzyme production, gene expression, etc.) as a result of a vitamin D stimulus. | 1.16E-02 | 2.89E-02 |
| REACTOME | REACTOME RIG I MDA5 MEDIATED INDUCTION OF IFN ALPHA BETA PATHWAYS | Genes involved in RIG-I/MDA5 mediated induction of IFN-alpha/beta pathways | 1.58E-02 | 3.93E-02 |
| REACTOME | REACTOME G1 PHASE | Genes involved in G1 Phase | 1.69E-02 | 2.93E-02 |
| GO | GO METHYL CPG BINDING | Interacting selectively and non-covalently with a methylated cytosine/guanine dinucleotide. | 2.04E-02 | 2.03E-03 |
| REACTOME | REACTOME TRAF6 MEDIATED INDUCTION OF TAK1 COMPLEX | Genes involved in TRAF6 mediated induction of TAK1 complex | 2.05E-02 | 4.03E-04 |
| GO | GO CELLULAR RESPONSE TO VITAMIN | Any process that results in a change in state or activity of a cell (in terms of movement, secretion, enzyme production, gene expression, etc.) as a result of a vitamin stimulus. | 2.44E-02 | 2.27E-02 |
| GO | GO POSITIVE REGULATION OF TRIGLYCERIDE METABOLIC PROCESS | Any process that modulates the frequency, rate or extent of the chemical reactions and pathways involving triglyceride, any triester of glycerol. | 2.54E-02 | 1.33E-02 |
| GO | GO FEEDING BEHAVIOR | Behavior associated with the intake of food. | 2.77E-02 | 3.46E-02 |
| GO | GO REVERSE CHOLESTEROL TRANSPORT | The directed movement of peripheral cell cholesterol, cholest-5-en-3-beta-ol, towards the liver for catabolism. | 2.87E-02 | 5.36E-05 |
| GO | GO CELLULAR RESPONSE TO NUTRIENT | Any process that results in a change in state or activity of a cell (in terms of movement, secretion, enzyme production, gene expression, etc.) as a result of a nutrient stimulus. | 2.91E-02 | 3.89E-02 |
| KEGG | KEGG VIBRIO CHOLERAE INFECTION | Cholera toxin (CTX) is one of the main virulence factors of Vibrio cholerae. Once secreted, CTX B-chain (CTXB) binds to ganglioside GM1 on the surface of the host's cells. After binding takes place, the entire CTX complex is carried from plasma membrane (PM) to endoplasmic reticulum (ER). In the ER, the A-chain (CTXA) is recognized by protein disulfide isomerase (PDI), unfolded, and delivered to the membrane where the membrane-associated ER-oxidase, Ero1, oxidizes PDI to release the CTXA into the protein-conducting channel, Sec61. CTXA is then retro-translocated to the cytosol and induces water and electrolyte secretion by increasing cAMP levels via adenylate cyclase (AC) to exert toxicity. Other than CTX, Vibrio cholerae generates several toxins that are perilous to eukaryotic cells. Zonula occludens toxin (ZOT) causes tight junction disruption through protein kinase C-dependent actin polymerization. RTX toxin (RtxA) causes actin depolymerization by covalently cross-linking actin monomers into dimers, trimers, and higher multimers. Vibrio cholerae cytolysin (VCC) is an important pore-forming toxin. The assembly of VCC anion channels in cells cause vacuolization and lysis. | 3.03E-02 | 3.88E-02 |
| REACTOME | REACTOME IRAK2 MEDIATED ACTIVATION OF TAK1 COMPLEX UPON TLR7 8 OR 9 STIMULATION | Genes involved in IRAK2 mediated activation of TAK1 complex upon TLR7/8 or 9 stimulation | 3.11E-02 | 9.16E-03 |
| REACTOME | REACTOME LIPOPROTEIN METABOLISM | Genes involved in Lipoprotein metabolism | 3.17E-02 | 2.11E-10 |
| REACTOME | REACTOME RIP MEDIATED NFKB ACTIVATION VIA DAI | Genes involved in RIP-mediated NFkB activation via DAI | 3.26E-02 | 3.37E-02 |
| GO | GO REGULATION OF ARF PROTEIN SIGNAL TRANSDUCTION | Any process that modulates the frequency, rate or extent of ARF protein signal transduction. | 3.30E-02 | 1.56E-02 |
| GO | GO LIPOPROTEIN PARTICLE RECEPTOR BINDING | Interacting selectively and non-covalently with a lipoprotein particle receptor. | 3.33E-02 | 1.39E-03 |
| GO | GO REGULATION OF CYCLIN DEPENDENT PROTEIN KINASE ACTIVITY | Any process that modulates the frequency, rate or extent of cyclin-dependent protein kinase activity. | 3.44E-02 | 2.79E-02 |
| GO | GO ARF GUANYL NUCLEOTIDE EXCHANGE FACTOR ACTIVITY | Stimulates the exchange of guanyl nucleotides associated with the GTPase ARF. Under normal cellular physiological conditions, the concentration of GTP is higher than that of GDP, favoring the replacement of GDP by GTP in association with the GTPase. | 3.65E-02 | 3.99E-02 |
| GO | GO LOW DENSITY LIPOPROTEIN PARTICLE RECEPTOR BINDING | Interacting selectively and non-covalently with a low-density lipoprotein particle, a lipoprotein particle that is rich in cholesterol esters and low in triglycerides, is typically composed of APOB100 and APOE, and has a density of 1.02-1.06 g/ml and a diameter of between 20-25 nm. | 3.76E-02 | 5.22E-03 |
| GO | GO REGULATION OF BLOOD VOLUME BY RENIN ANGIOTENSIN | The process in which the renin-angiotensin system controls the rate of fluid intake and output into the blood. | 3.87E-02 | 1.54E-02 |
| GO | GO 90S PRERIBOSOME | A large ribonucleoprotein complex considered to be the earliest preribosomal complex. In S. cerevisiae, it has a size of 90S and consists of the 35S pre-rRNA, early-associating ribosomal proteins most of which are part of the small ribosomal subunit, the U3 snoRNA and associated proteins. | 3.91E-02 | 6.67E-03 |
| REACTOME | REACTOME TRIF MEDIATED TLR3 SIGNALING | Genes involved in TRIF mediated TLR3 signaling | 3.91E-02 | 2.38E-02 |
| GO | GO INTRINSIC COMPONENT OF MITOCHONDRIAL MEMBRANE | The component of the mitochondrial membrane consisting of the gene products and protein complexes having either part of their peptide sequence embedded in the hydrophobic region of the membrane or some other covalently attached group such as a GPI anchor that is similarly embedded in the membrane. | 4.20E-02 | 3.71E-03 |
| REACTOME | REACTOME NFKB AND MAP KINASES ACTIVATION MEDIATED BY TLR4 SIGNALING REPERTOIRE | Genes involved in NFkB and MAP kinases activation mediated by TLR4 signaling repertoire | 4.22E-02 | 3.47E-02 |
| GO | GO NEGATIVE REGULATION OF HORMONE SECRETION | Any process that stops, prevents, or reduces the frequency, rate or extent of the regulated release of a hormone from a cell. | 4.75E-02 | 2.69E-02 |
| BIOCARTA | BIOCARTA PYK2 PATHWAY | This diagram is a compilation of Pyk2 effort cascades. In specific cell types the receptor and effoectors will vary. Binding of a transmembrane receptor triggers the activation of Ca2+ signaling and PKC. The signal is then transmitted to Pyk2 and further to the small G protein Rac1. In turn, Rac1 initiatates the JNK cascade, starting with PAK follwed by MEKK1, SEK1, and JNK. JNK activation causes induction of c-Jun gene binding. Pyk2 stimulation has also been shown to activate MKK3 leading to activation of p38. The other major mitogen activated kinase cascade for ERK1/2 is stimulated via RAS, RAF and MEKK1/2. | 4.79E-02 | 4.97E-02 |

1. P values derived from pathway-based analysis in our study participants;
2. P values derived from pathway-based analysis in existing *in silico* meta-GWAS dataset.

**Supplementary Fig. 1.** Summary of sample and marker quality control within the two study cohort.

**Supplementary Fig. 2.** Principal component analysis. The first two principal components for each individual were plotted: (A) 1,669 cases from ACS Genetic Study, together with Asian (JPT, CHB and CHS), African (YRI), and European (CEU) populations from 1000 Genome Project; (B) 1,669 cases from ACS Genetic Study and 1,935 controls from PUUMA-MI study together with Asian (JPT, CHB and CHS), African (YRI), and European (CEU) populations from 1000 Genome Project; and (C) the population structure of the cases and controls.

A

**
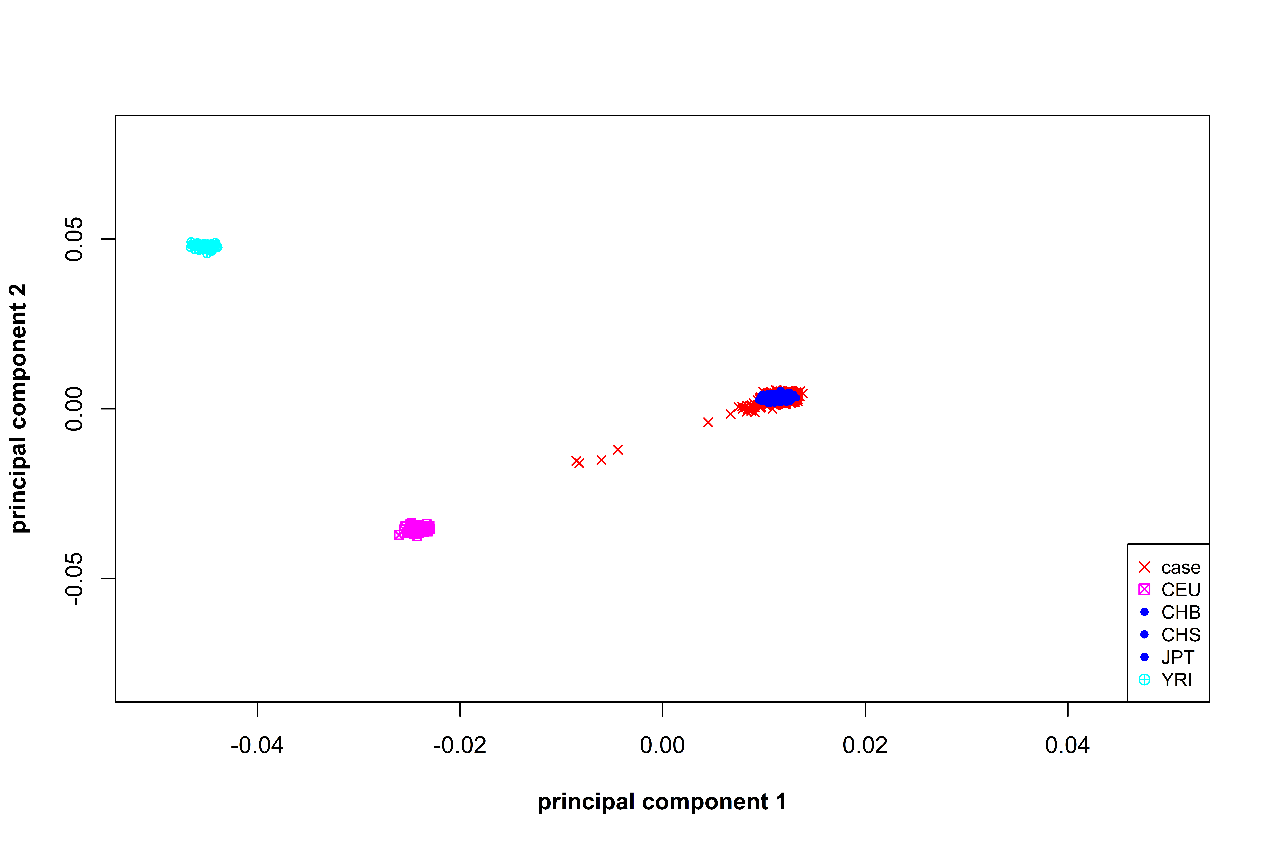
**

B

**
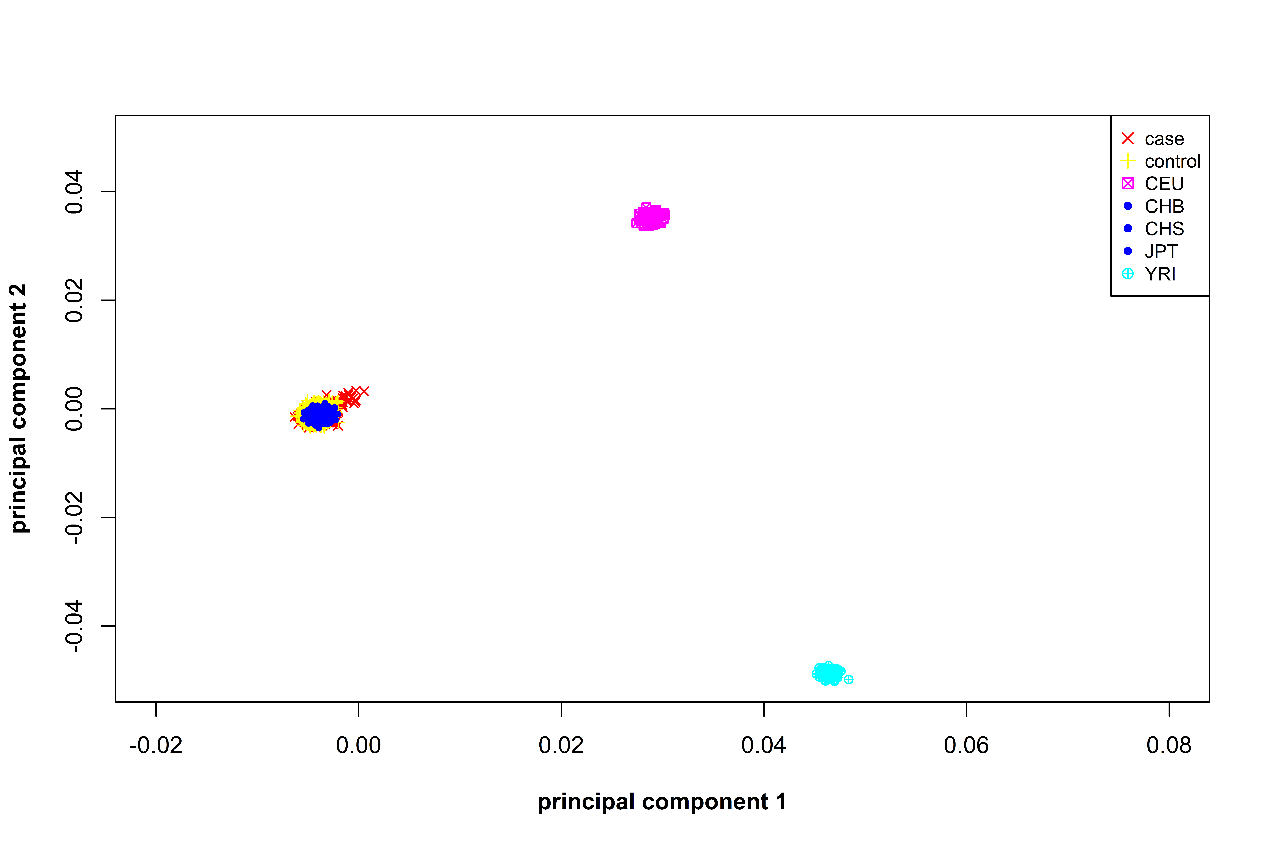
**

C


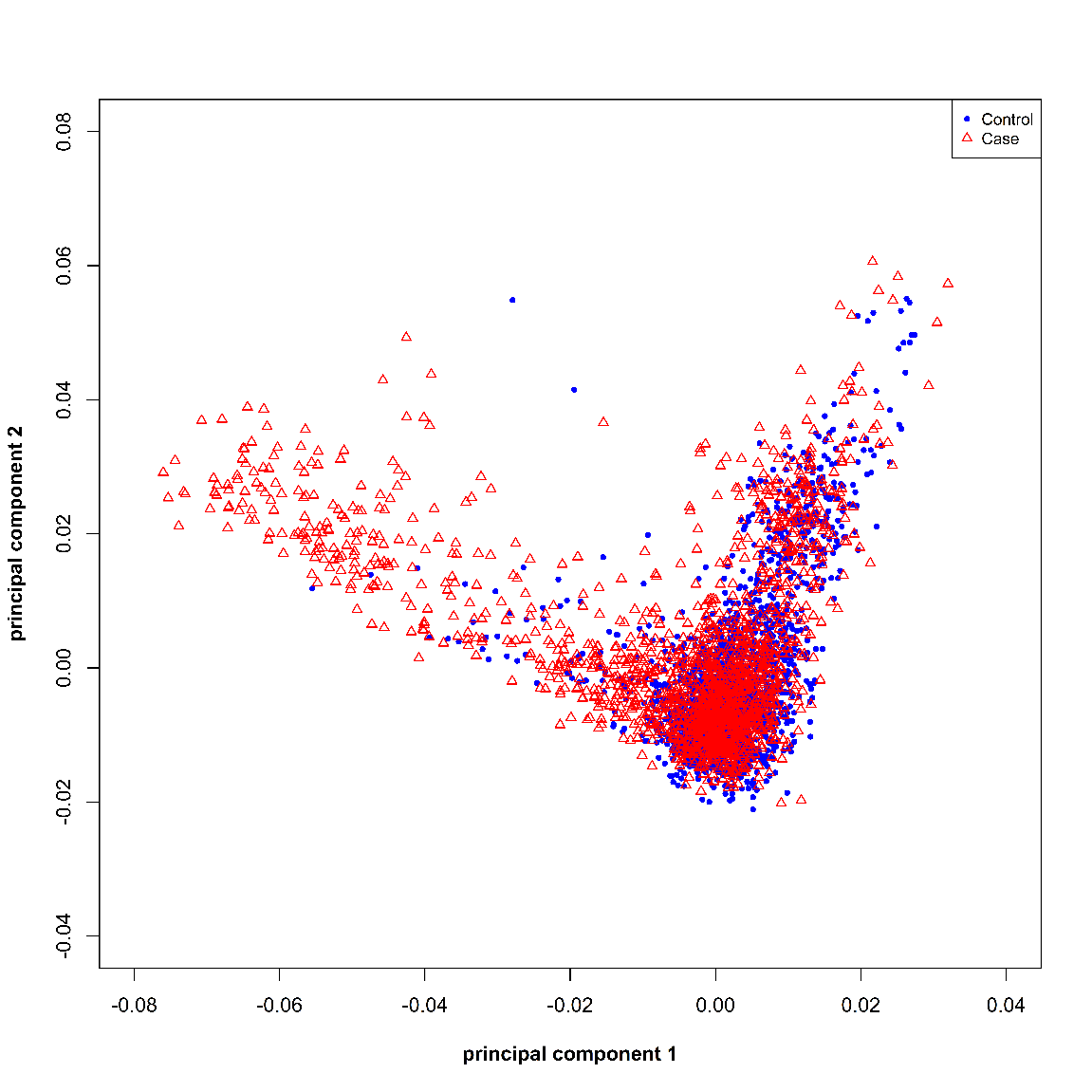


**Supplementary Fig. 3.** Risk distribution of participants from PUUMA study according to their 10-year ASCVD risk.


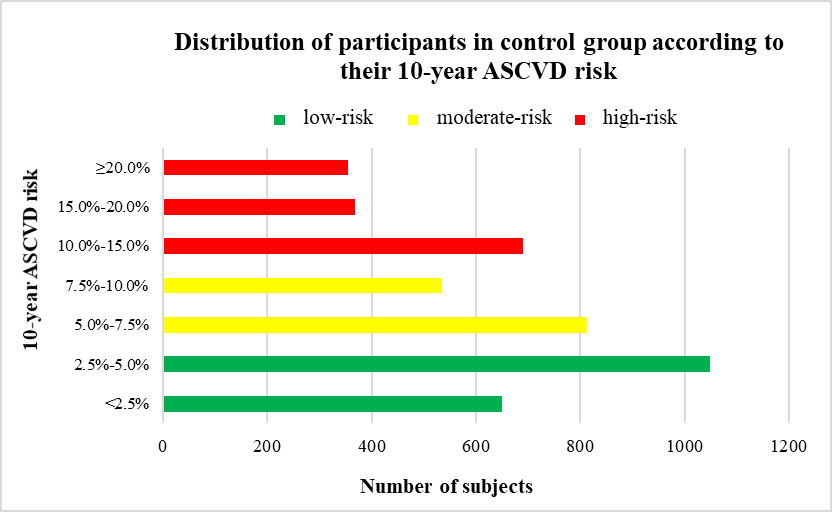


**Supplementary Fig. 4.** Quantile-quantile plot of single-variant analysis results of acute coronary syndrome (λ_GC_ = 1.000).


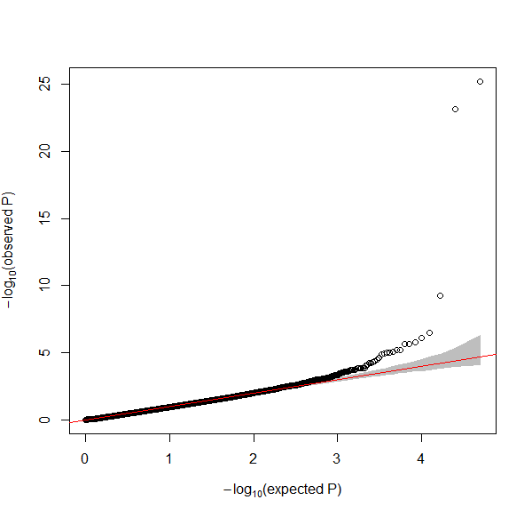


**Supplementary Fig. 5.** Manhattan plot for associations between genetic variants and ACS risk. The association analyses were based on 1,669 ACS cases and 1,935 controls, which were selected according to China-PAR risk prediction model for 10-year risk ASCVD. *P* values are two-sided and were calculated by an addictive model in logistic regression analysis adjusted for age, gender, and the first two principal components. Red line indicated the exome-chip-wide significance threshold (*P* < 1×10^-6^) and blue line indicated the (*P* < 1.0×10^-4^).


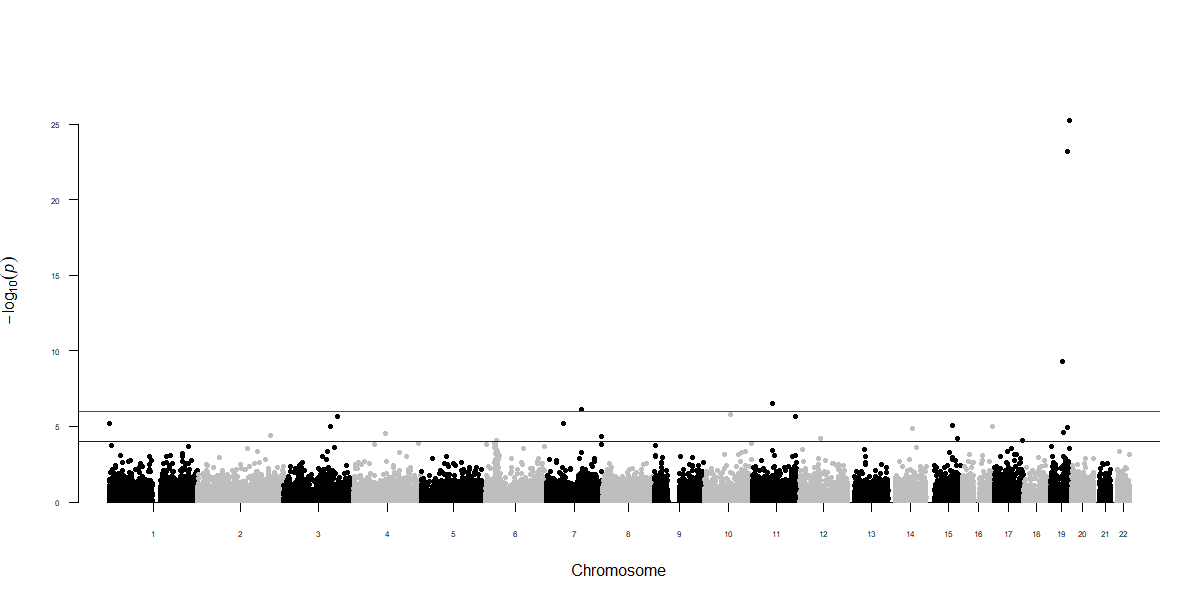


**Supplementary Fig. 6.** The differential expressions of *ZNF655* based on GSE66360 dataset. The expression levels of *ZNF655* (log_2_ FC = -1.4, *P* = 2.4×10^-9^) were significantly downregulated in MI cases.


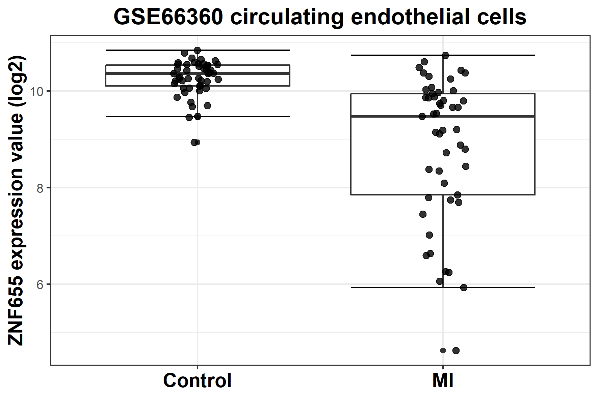

Supplement: Supplementary file 2 [file Data_Sheet_2.docx]
